# Visual dictionaries as intermediate features in the human brain

**DOI:** 10.3389/fncom.2014.00168

**Published:** 2015-01-15

**Authors:** Kandan Ramakrishnan, H. Steven Scholte, Iris I. A. Groen, Arnold W. M. Smeulders, Sennay Ghebreab

**Affiliations:** ^1^Intelligent Systems Lab Amsterdam, Institute of Informatics, University of AmsterdamAmsterdam, Netherlands; ^2^Cognitive Neuroscience Group, Department of Psychology, University of AmsterdamAmsterdam, Netherlands

**Keywords:** visual perception, fMRI, low and intermediate features, HMAX, bag of words, representation similarity analysis

## Abstract

The human visual system is assumed to transform low level visual features to object and scene representations via features of intermediate complexity. How the brain computationally represents intermediate features is still unclear. To further elucidate this, we compared the biologically plausible HMAX model and Bag of Words (BoW) model from computer vision. Both these computational models use visual dictionaries, candidate features of intermediate complexity, to represent visual scenes, and the models have been proven effective in automatic object and scene recognition. These models however differ in the computation of visual dictionaries and pooling techniques. We investigated where in the brain and to what extent human fMRI responses to short video can be accounted for by multiple hierarchical levels of the HMAX and BoW models. Brain activity of 20 subjects obtained while viewing a short video clip was analyzed voxel-wise using a distance-based variation partitioning method. Results revealed that both HMAX and BoW explain a significant amount of brain activity in early visual regions V1, V2, and V3. However, BoW exhibits more consistency across subjects in accounting for brain activity compared to HMAX. Furthermore, visual dictionary representations by HMAX and BoW explain significantly some brain activity in higher areas which are believed to process intermediate features. Overall our results indicate that, although both HMAX and BoW account for activity in the human visual system, the BoW seems to more faithfully represent neural responses in low and intermediate level visual areas of the brain.

## 1. Introduction

The human visual system transforms low-level features in the visual input into high-level concepts such as objects and scene categories. Visual recognition has been typically viewed as a bottom-up hierarchy in which information is processed sequentially with increasing complexities, where lower-level cortical processors, such as the primary visual cortex, are at the bottom of the processing hierarchy and higher-level cortical processors, such as the inferotemporal cortex (IT), are at the top, where recognition is facilitated (Bar, 2003). Much is known about the computation in the earliest processing stages, which involve the retina, lateral geniculate nucleus (LGN) and primary visual cortex (V1). These areas extract simple local features such as blobs, oriented lines, edges and color from the visual input. However, there remain many questions on how such low-level features are transformed into high-level object and scene percepts.

One possibility is that the human visual system transforms low-level features into object and scene representations via an intermediate step (Riesenhuber and Poggio, 1999). After extraction of low-level features in areas such as V1, moderately complex features are created in areas V4 and the adjacent region TO. Then partial or complete object views are represented in anterior regions of inferotemporal (IT) cortex (Tanaka, 1997). It has been suggested that such intermediate features along the ventral visual pathway are important for object and scene representation (Logothetis and Sheinberg, 1996).

Previous studies have provided some evidence of what intermediate features might entail. In Tanaka (1996) it has been shown that cells in the V4/IT region respond selectively to complex features such as simple patterns and shapes. Similarly, Hung et al. (2012) identified contour selectivity for individual neurons in the primate visual cortex and found that most contour-selective neurons in V4 and IT each encoded some subset of the parameter space and that a small collection of the contour-selective units were sufficient to capture the overall appearance of an object. Together these findings suggest that intermediate features capture object information encoded within the human ventral pathway.

In an attempt to answer the question of intermediate features underlying neural object representation, Leeds et al. (2013) compared five different computational models of visual representation against human brain activity to object stimuli. They found that the Bag of Words (BoW) model was most strongly correlated with brain activity associated with midlevel perception. These results were based on fMRI data from 5 subjects. Recently Yamins et al. (2014) used a wider set of models including HMAX and BoW against neural responses from two monkeys in IT and V4.

HMAX (Riesenhuber and Poggio, 1999) and BoW (Csurka et al., 2004) models represent scenes in a hierarchical manner transforming low level features to high level concepts. HMAX is a model for the initial feedforward stage of object recognition in the ventral visual pathway. It extends the idea of simple cells (detecting oriented edges) and complex cells (detecting oriented edges with spatial invariance) by forming a hierarchy in which alternate template matching and max pooling operations progressively build up feature selectivity and invariance to position and scale. HMAX is thus a simple and elegant model used by many neuroscientists to describe feedforward visual processing. In computer vision, different algorithms are used for object and scene representation. The commonly used model in computer vision is BoW which performs very well on large TRECvid (Smeaton et al., 2006) and PASCAL (Everingham et al., 2010) datasets, in some cases even approaching human classification performance (Parikh and Zitnick, 2010). The key idea behind this model is to quantize local Scale Invariant Feature Transform (SIFT) features (Lowe, 2004) into visual words (Jurie and Bill, 2005), features of intermediate complexity, and then to represent an image by a histogram of visual words. To further understand the nature of intermediate features underlying scene perception, we test these two computational models against human brain activity while subjects view a movie of natural scenes.

Although HMAX and BoW are different models they both rely on the concept of visual dictionaries to represent scenes. In HMAX after the initial convolution and pooling stage, template patches are learnt from responses of the pooling layer (from a dataset of images) which are used as visual dictionaries. In the BoW model, clustering of SIFT features forms the visual dictionary. In both the models, visual dictionaries are medium size image patches that are informative and at the same time distinctive. They can be thought of, as features of intermediate complexity. This comparison of different computational approaches to visual dictionaries might provide further insight about the representation of intermediate features in the human brain.

In this work we test two layers of the HMAX and BoW models against human brain activity. We show 20 subjects a 11-min video of dynamic natural scenes and record their fMRI activity while watching the video. We use dynamic scenes instead of static scenes because they are more realistic, and because they may evoke brain responses that allow for a better acquisition of neural processes in the visual areas of the brain (Hasson et al., 2004). Furthermore, the use of a relatively large pool of subjects allows us to compare computational models in terms of their consistency in explaining brain activity. The fMRI data is compared to HMAX and BoW models. For the HMAX model we test how Gabor and visual dictionary representation of an image explain brain activity. Similarly for BoW, we test how SIFT and visual dictionary explain brain activity. If the models are good representations of intermediate features in the human brain, they should account for brain activity across multiple subjects.

Testing hierarchical models of vision against brain activity is challenging for two reasons. First, computational and neural representations of visual stimuli are of different nature but both very high-dimensional. Second the different hierarchical levels of the models need to be dissociated properly in order to determine how brain activity is accounted by each of the individual hiearchical levels of the model. This cannot be done easily in standard multivariate neuroimaging analysis. We address the first challenge by using dissimilarity matrices (Kriegeskorte et al., 2008) that capture computational and neural distances between any pair of stimuli. The second is resolved by applying a novel technique, variation partitioning (Peres-Neto et al., 2006) on the dissimilarity matrices. This enables us to compute the unique contributions of the hierarchical layers of HMAX and BoW models in explaining neural activity. Distance based variation partitioning has been successfully used in ecological and evolutionary studies, and will be applied here to fMRI data. This will enable us to establish correspondence between computational vision models, their different hierarchical layers and fMRI brain activity.

## 2. Materials and methods

### 2.1. Computational models

**Figure 1:**
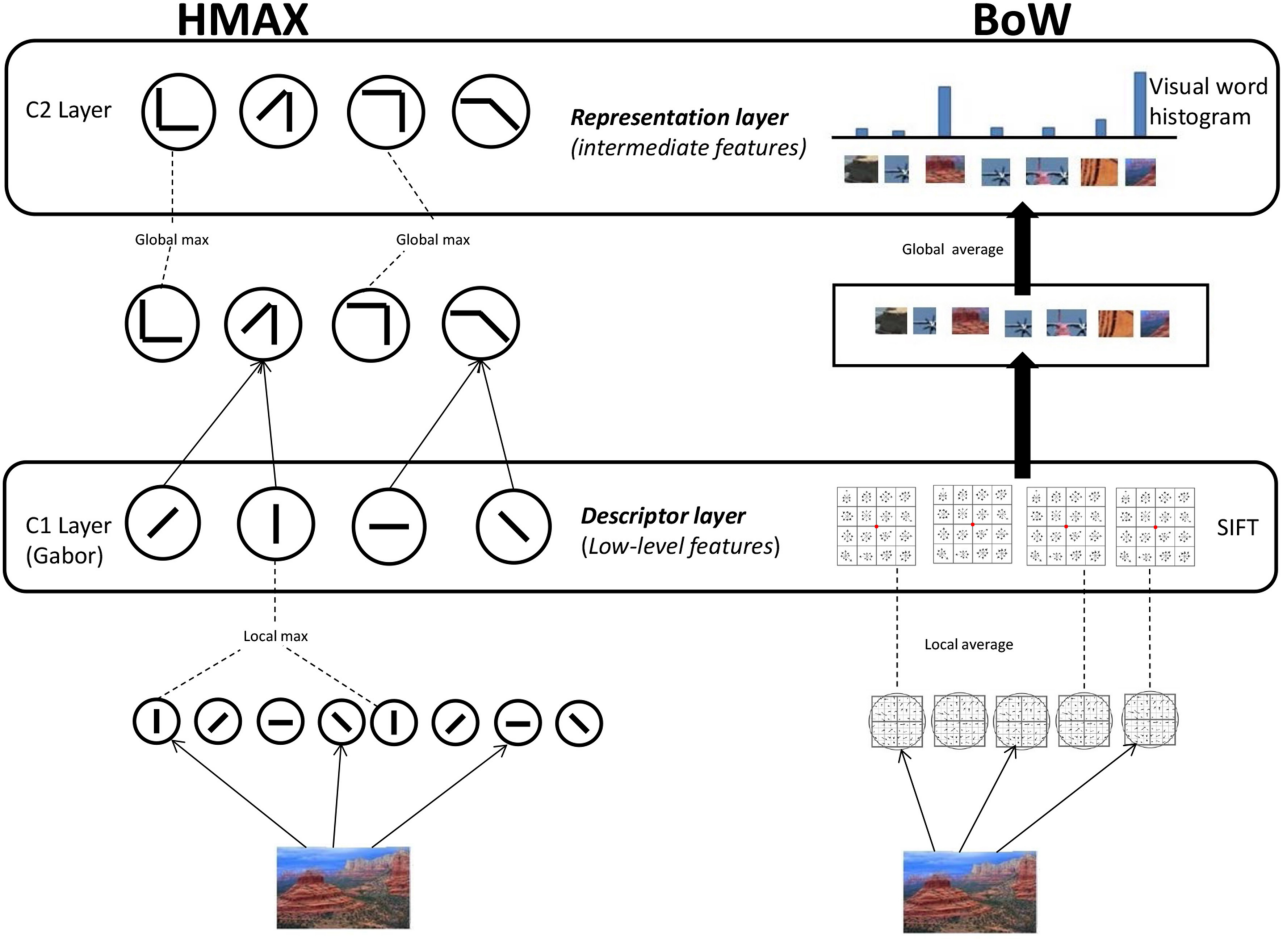
**Computational models**. **HMAX model:** Gabor filters of 4 orientations and 10 different scales are convolved in the S1 Layer. The responses are pooled to form the C1 Layer. For the S2 Layer random samples of the pooled responses from the C1 layer to the PASCAL dataset of images is used to form the visual dictionary of dimension 4096. These template patches are detected from the responses to form the S2 Layer. A global max pooling operation is done for the final C2 Layer which is of dimension 4096. **BoW model:** SIFT features are extracted densely over the image. Visual dictionary of dimension 4000 is learnt by kmeans clustering on SIFT features extracted PASCAL dataset images. Each SIFT descriptor from an image is encoded to the nearest element of visual dictionary. Average pooling is done to form the 4000 dimension visual dictionary representation.

We use HMAX and BoW computational models to represent images at the different hierarchical levels. For the HMAX model we compute the Gabor representation at the first level and the visual dictionary representation at the second level (Figure 1). Similarly for the BoW model we compute the SIFT representation and visual dictionary representation. It is important to note that HMAX and BoW models refer to the entire hierarchical model combining low level feature and visual dictionary.

#### 2.1.1. HMAX model

We use the HMAX model (Mutch and Lowe, 2008), where features are computed hierarchically in layers: an initial image layer and four subsequent layers, each built from the previous layer by alternating template matching and max pooling operations as seen in Figure 1. In the first step, the greyscale version of an image is downsampled and a image pyramid of 10 scales is created. Gabor filters of four orientations are convolved over the image at different positions and scales in the next step, the S1 layer. Then in the C1 layer, the Gabor responses are maximally pooled over 10 × 10 × 2 regions of the responses from the previous layer (the max filter is a pyramid). The Gabor representation of an image *I* is denoted by the vector **f**_*gabor*_.

In the next step, template matching is performed between the patch of C1 units centered at every position/scale and each of *P* prototype patches. These *P* = 4096 prototype patches are learned as done in Mutch and Lowe (2008) by randomly sampling patches from the C1 layer. We use images from the PASCAL VOC 2007 dataset (Smeaton et al., 2006) to sample the prototypes for the dictionary. In the last layer, a *P* dimensional feature is created by maximally pooling over all scales and orientations to one of the models *P* patches from the visual dictionary. This results in a visual dictionary representation of image *I* denoted by the vector **f**_*vdhmax*_ = [*h*_1_ … *h_P_*] where each dimension *h_p_* represents the max response of the dictionary elements convolved over the output of the C1 layer.

#### 2.1.2. BoW model

The first step in the BoW model (Figure 1) is extraction of SIFT descriptors (Lowe, 2004) from the image. SIFT combines a scale invariant region detector and a descriptor based on the gradient distribution in the detected regions. The descriptor is represented by a 3D histogram of gradient locations and orientations weighted by the gradient magnitude. The quantization of gradient locations and orientations makes the descriptor robust to small geometric distortions and small errors in the region detection. SIFT feature is a 128 dimensional vector which is computed densely over the image. Here the SIFT representation of an image *I* is obtained by concatenating all the SIFT features over the image. It is denoted by the vector **f**_*sift*_.

Secondly, a dictionary of visual words (Csurka et al., 2004) is learned from a set of scenes independent of the scenes in the stimuli video. We use k-means clustering to identify cluster centers **c**_*m*_ = **c**_1_, …, **c**_*M*_ in SIFT space, where *m* = 1, …, *M* denotes the number of visual words. We use the PASCAL VOC 2007 (Smeaton et al., 2006) dataset to create a codebook of dimension *M* = 4000.

The SIFT features of a new image are quantized (assigned to the nearest visual word) to a element in the visual dictionary and the image is represented by counting the occurrences of all words. This results for image *I* in the visual dictionary representation **f**_*vdbow*_ = [*h*_1_ … *h_M_*] where each bin *h_m_* indicates the frequency(number of times) the visual word **c**_*m*_ is present in the image.

### 2.2. Representational dissimilarity matrices

**Figure 2:**
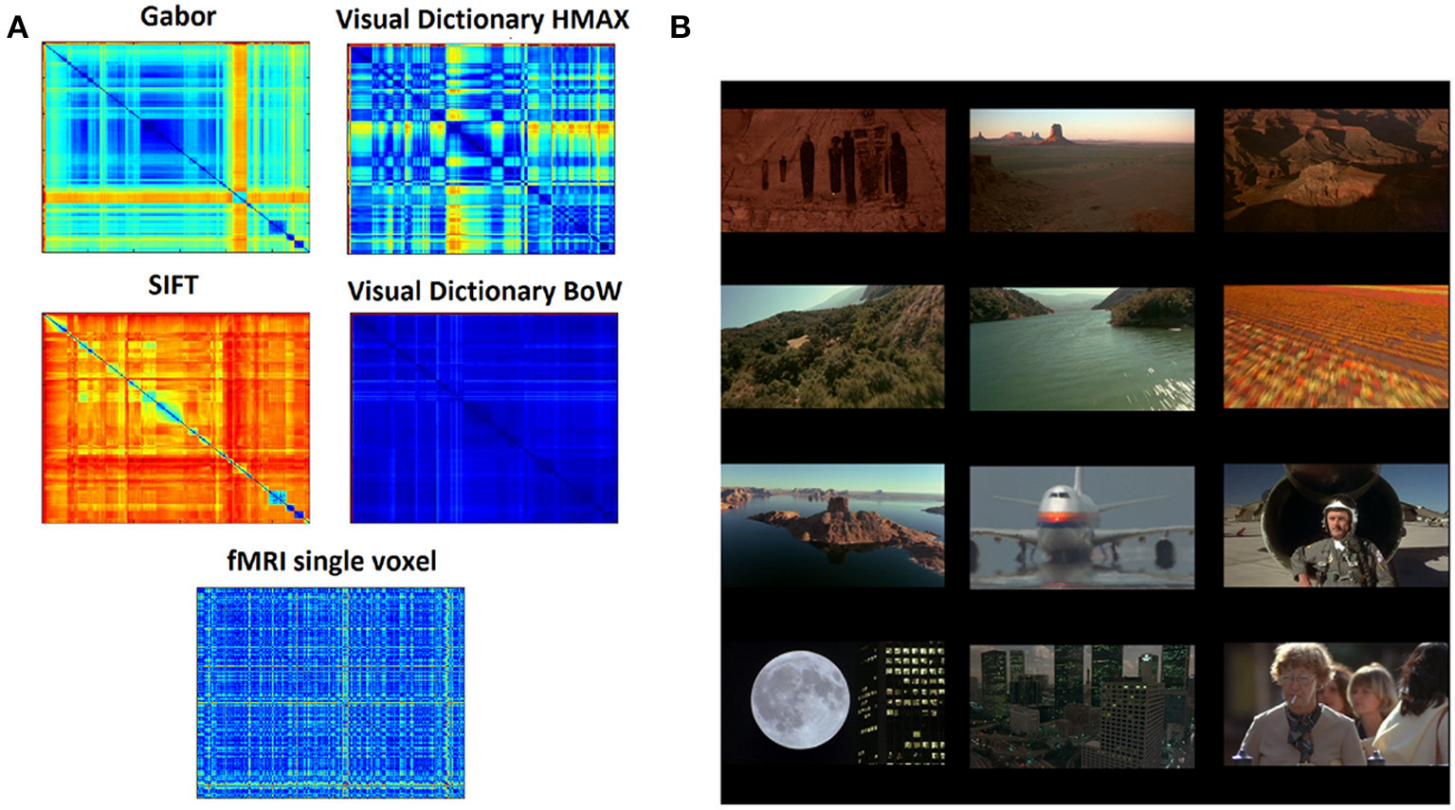
**Data and representations (A) Dissimilarity matrices computed for the different hierarchical levels of HMAX and BoW using pairwise distance between all the scenes from the stimuli resulting in a 290 × 290 matrix**. Similarly the dissimilarity matrix computed for the fMRI brain responses where each element is the distance in multivariate voxel responses to any image pair resulting in a 290 × 290 matrix. **(B)** Example images (frames) from the 11 min video stimuli that was used in the fMRI study. There are totally 290 scenes representing a wide variety of scenes, ranging from natural to man-made.

A representational dissimilarity matrix (Kriegeskorte et al., 2008) (RDM) *F* is computed separately for each of the image representations. The elements in this matrix are the Euclidean distance between the representations of pairs of images. Thus, *F*_*gabor*_, *F_vdhmax_*, *F_sift_*, and *F_vdbow_* are dissimilarity matrices for the different representations respectively. Figure 2A shows the 290 × 290 dissimilarity matrices for 290 images (frames) from the video stimulus used in this study.

### 2.3. Stimuli

An 11-min video track consisting of about 20 different dynamic scenes was used for this study. The scenes were taken from the movie Koyaanisqatsi: Life Out of Balance and consisted primarily of slow motion and time-lapse footage of cities and many natural landscapes across the United States as in Figure 2B.

The movie Life Out of Balance was chosen as a stimulus because it contained all kinds of scenes we encounter in our daily live with no human emotional content or specific storyline, from natural (e.g., forest) to more man made scenes (e.g., streets). In addition the movie exhibits different motion elements such as zooming, scaling, luminance etc. In this respect, the movie is rich in its underlying low-level properties such as spatial frequency and color.

### 2.4. Subjects

The fMRI data of the video stimuli was collected for over 500 subjects, from which 20 were randomly sampled for this study. Subjects were not assigned with any specific tasks when watching. They watched the video track passively one time each. The experiment was approved by the ethical committee of the University of Amsterdam and all participants gave written informed consent prior to participation. They were rewarded for participation with either study credits or financial compensation.

### 2.5. fMRI

We recorded 290 volumes of BOLD-MRI (GE-EPI, 192^2^ mm, 42 slices, voxel size of 3 × 3 × 3.3, TR 2200 ms, TE 27.63 ms, SENSE 2, FA 90°C) using a 3T Philips Achieve scanner with a 32 channel headcoil. A high-resolution T1-weighted image (TR, 8.141 ms; TE, 3.74 ms; FOV, 256 × 256 × 160 mm) was collected for registration purposes. Stimuli were backward-projected onto a screen that was viewed through a mirror attached to the head-coil.

### 2.6. fMRI preprocessing

FEAT (fMRI Expert Analysis Tool) version 5.0, part of FSL (Jenkinson et al., 2012) was used to analyze the fMRI data. Preprocessing steps included slice-time correction, motion correction, high-pass filtering in the temporal domain (σ = 100 s), spatially filtered with a FWHM of 5 mm and prewhitened (Woolrich et al., 2001). Data was transformed using an ICA and we subsequently, automatically identified artifacts using the FIX algorithm (Salimi-Khorshidi et al., 2014). Structural images were coregistered to the functional images and transformed to MNI standard space (Montreal Neurological Institute) using FLIRT (FMRIB's Linear Image Registration Tool; FSL). The resulting normalization parameters were applied to the functional images. The data was transformed into standard space for cross-participant analyses, so that the same voxels and features were used across subjects.

These 290 image frames and volumes were used to establish a relation between the two computational models and BOLD responses. Although in this approach the haemodynamic response might be influenced by other image frames, we expect this influence to be limited because the video is slowly changing without any abrupt variations. In addition, BOLD responses are intrinsically slow and develop over a period of up to 20 s. Still they summate linearly reasonably well (Buckner, 1998) and also match the timecourse in typical scenes which develop over multiple seconds. This also probably explains the power of BOLD-MRI in decoding the content of movies (Nishimoto et al., 2011) and indicates it is possible to compare different models of information processing on the basis of MRI volumes.

### 2.7. Variation partitioning

A 3 × 3 × 3 searchlight cube is centered at each voxel in the brain and BOLD responses within the cube to each of the 290 still images compared against each other. This results for each subject and for each voxel in a 290 × 290 dissimilarity matrix *Y*. Each element in the *Y* matrix is the pairwise distance of the 27 dimensional (from the searchlight cube) multivariate voxel responses to any image pair. As a distance measure Cityblock is taken. We now perform variation partitioning voxel-wise (each voxel described by its searchlight cube) for all the voxels across all subjects.

Variation partitioning (Peres-Neto et al., 2006) for the HMAX model is done by a series of multiple regression, producing fractions of explained variation *R*^2^_*gabor*_ (unique to gabor representation), *R*^2^_*gaborvdhmax*_ (common to both gabor and visual dictionary representation) and *R*^2^_*vdhmax*_ (unique to visual dictionary). First the multiple regression of Y against *F_gabor_* and *F_vdhmax_* together is computed, where Y denotes the fMRI dissimilarity matrix, and *F_gabor_* and *F_vdhmax_* the Gabor and visual dictionary dissimilarity matrices respectively. The corresponding *R*^2^_*hmax*_ measures the total fraction of explained variation, which is the sum of the fractions of variation *R*^2^_*gabor*_, *R*^2^_*gaborvdhmax*_, and *R*^2^_*vdhmax*_. Then the multiple regression of Y against *F_gabor_* is computed. The corresponding *R*^2^_*gabor*+*gaborvdhmax*_ measure is the sum of the fractions *R*^2^_*gabor*_ and *R*^2^_*gaborvdhmax*_. In the next step, the multiple regression of Y against *F_vdhmax_* is obtained, with corresponding *R*^2^_*vdhmax*+*gaborvdhmax*_ being the sum of the fractions of variation *R*^2^_*gaborvdhmax*_ and *R*^2^_*vdhmax*_. The fraction of variation uniquely explained by the Gabor dissimilarity matrix is computed by substraction: *R*^2^_*gabor*_ = *R*^2^_*hmax*_ - *R*^2^_*vdhmax*+*gaborvdhmax*_. Similarly, variation uniquely explained by visual dictionary dissimilarity matrix is: *R*^2^_*vdhmax*_ = *R*^2^_*hmax*_ - *R*^2^_*gabor*+*gaborvdhmax*_. The residual fraction may be computed by: 1 − (*R*^2^_*gabor*_ + *R*^2^_*gaborvdhmax*_ + R^2^_*vdhmax*_).

**Figure 3:**
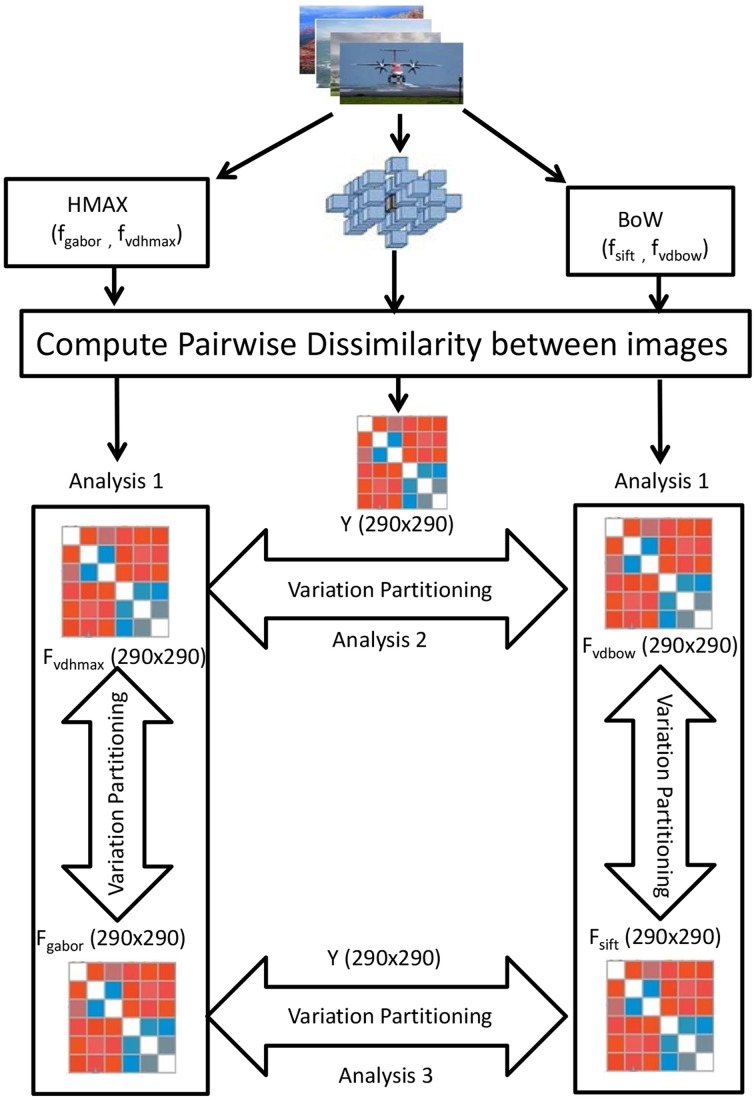
**Visualization of the variation partitioning on the RDMs obtained from the 290 images of the ID1000 stimuli**. For the HMAX model we obtain a 290 × 290 Gabor dissimilarity matrix(*F_gabor_*) and visual dictionary dissimilarity matrix(*F_vdhmax_*) using pairwise image distances. Similarly for BoW, we obtain 290 × 290 SIFT dissimilarity matrix(*F_sift_*) and visual dictionary matrix(*F_vdbow_*). Then variation partitioning is applied at each of the hierarchical level and across the hierarchical levels on the 290 × 290 fMRI dissimilarity matrix(Y).

Exactly the same steps of computation are taken to determine the fraction of variation uniquely explained by the SIFT dissimilarity matrix, the fraction explained by BoW visual dictionary dissimilarity matrix, and by the combination of both the SIFT and visual dictionary dissimilarity matrices as shown in Figure 3.

We also compare the models at their respective hierarchical levels. At the first level, Gabor and SIFT dissimilarity matrices are used to explain brain activity *Y*. Similarly at the level of visual dictionaries, we compare how HMAX and BoW visual dictionary dissimilarity matrices explain *Y*.

Note that these *R*^2^ statistics are the canonical equivalent of the regression coefficient of determination, *R*^2^ (Peres-Neto et al., 2006). They can interpreted as the proportion of the variance in the dependent variable that is predictable from the independent variable.

A permutation test (1000 times) determines the statistical significance (*p* value) of the fractions that we obtain for each voxel by variation partitioning. To account for the multiple comparison problem, we perform cluster size correction and only report here clusters of voxels that survive the statistical thresholding at *p* < 0.05 and have a minimum cluster size of 25 voxels. We determine the minimum cluster size by calculating the probability of a false positive from the frequency count of cluster sizes within the entire volume, using a Monte Carlo simulation (Ward, 2000).

## 3. Results

### 3.1. Comparing full models: intersubject consistency

Using distance-based variation partitioning for each subject we dissociate the explained variation of the HMAX model into unique contributions of Gabor *R*^2^_*gabor*_ and visual dictionary representation *R*^2^_*vdhmax*_. The total explained variation by HMAX model is given by the combination of *R*^2^_*gabor*_ and *R*^2^_*vdhmax*_. We do the same for the BoW model, based on SIFT *R*^2^_*sift*_ and visual dictionary representation *R*^2^_*vdbow*_. HMAX and BoW models refer to the entire hierarchical model combining low level feature and visual dictionary. Cluster size correction (*p* < 0.05 and minimal cluster size of 25 voxels) was performed to solve for the multiple comparison problem.

To test whether our results are consistent across subjects, for each voxel we counted the number subjects for which brain activity was explained significantly by the HMAX and BoW models. A spatial version of the chi-square statistic (Rogerson, 1999) was subsequently applied to determine whether the observed frequency at a particular voxel deviated significantly from the expected value (the average number of subjects across all voxels).

**Figure 4:**
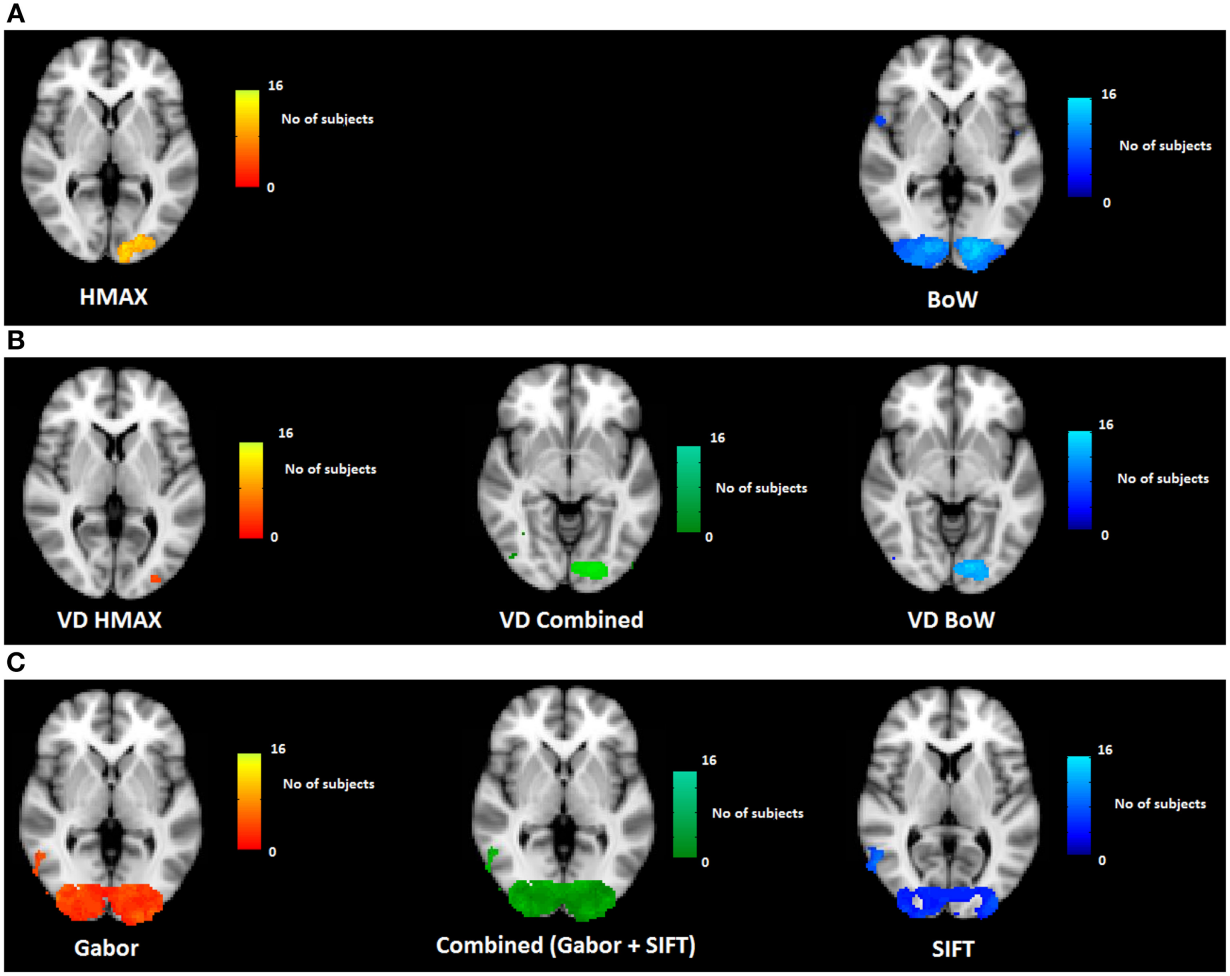
**Visualization of across subject consistency at each voxel for the complete HMAX and BoW models and their individual components**. To find consistency across subjects, first significant voxel clusters are determined subject wise and then a spatial frequency count is performed on detected clusters across subjects. **(A)** Across subjects consistency for HMAX and BoW model based on voxel clusters and the spatial chi-square statistic (Analysis 1 in Figure 3). **(B)** Across subjects consistency for visual dictionaries from HMAX (VD HMAX), BoW (VD BoW) and their combination (Analysis 2 in Figure 3). **(C)** Across subjects consistency for Gabor, SIFT and their combination (Analysis 3 in Figure 3).

Figure 4A shows how consistently across subjects, HMAX and BoW models account for brain activity. We observe that the HMAX model explains brain activity in areas V2 and V3 consistently across subjects. In these areas the HMAX model explains brain activity in overlapping voxels for 16 out of 20 subjects. In contrast, the BoW model accounts for brain activity across wider and bilateral regions including V1, V2 and V3. Most consistency is found at the left V3 and V4 regions, where for 14 out of 20 subjects, the BoW model was relevant in explaining brain activity. This difference in the number of subjects is not significant however the extent of the voxels is much more for BoW than HMAX.

Both HMAX and BoW models use low level features (Gabor filters and histogram of orientations) as their first step of computation. This is explicitly modeled and tested in our study (low-level feature representation in Figure 1). This explains why low level visual regions such as V1 and V2 emerge in our results. Interestingly, however, the BoW model also accounts for brain activity in regions higher up in the visual system such as V4 and LO (lateral occipital cortex). These regions are hypothesized to process intermediate features. This suggests that while both models appropriately represent low-level features, the transformation of these features to intermediate features is better modeled by BoW. Figure 1 in Supplementary section shows for each individual subject the explained variation of the two representational levels in both the models.

We observe that for the HMAX model the combination of hierarchies provide 5% of additional explanation compared to the maximum explaining hierarchical level. The two levels of the BoW together additionally account for 8% of the variation in brain activity. A *t*-test on the two distributions of additional explained variations show a significant difference (*p* < 0.0001). Thus, in both models, but more strongly in BoW, the aggregation of low level features into visual dictionaries describes brain activity, not captured by individual hierarchical levels. Thus, the aggregation of low level features into visual dictionaries provide additional value to account for brain activity. The hierarchical levels in BoW contribute slightly more to the explained brain activity as compared to the hierarchical levels from HMAX.

### 3.2. Comparing visual dictionaries: intersubject consistency

We tested the two visual dictionary representations against each other. As before, we use variation partitioning on the visual dictionary dissimilarity matrices from HMAX and BoW to explain *Y*. For each voxel we counted the number of subjects for which brain activity was explained significantly by the visual dictionary from HMAX and BoW models. A spatial version of the chi-square statistic (Rogerson, 1999) was applied to determine whether the observed frequency at a particular voxel deviated significantly from the expected value (the average count across all voxels).

Figure 4B shows the across subject consistency of visual dictionaries from HMAX and BoW models (*p* < 0.05, cluster size correction). We observe for the HMAX visual dictionary representation that consistency across subjects occurs in few voxels in area V4. In contrast the visual dictionary representation of the BoW model explains brain activity in areas V3 and V4 for 14 out of 20 subjects. The combination of visual dictionary representation explain brain activity for 14 out of 20 subjects in areas V3 and V4.

The visual dictionary representation from the BoW model has a much higher across subject consistency than the HMAX model. In addition the results of the combined model are similar to those of the BoW visual dictionary representations, suggesting that the HMAX visual word representation adds little to the BoW representation in terms of accounting for brain activity. Moreover, the BoW visual dictionary representation is localized in an area V4 that is hypothesized to compute intermediate features. Altogether, these results suggest that the BoW model provides a better representation for visual dictionaries, compared to the HMAX model. Single subject results confirming consistency across subjects can be found in the Supplementary section.

### 3.3. Comparing low-level feature representations: intersubject consistency

We tested Gabor and SIFT representation against each other. As before, we use variation partitioning on Gabor and SIFT representations to explain *Y*. For each voxel we counted the number subjects for which brain activity was explained significantly. The spatial version of the chi-square statistic was applied to determine whether the observed frequency at a particular voxel deviated significantly from the expected value (the average count across all voxels).

Figure 4C shows the across subject consistency of Gabor and SIFT representations (*p* < 0.05, cluster size correction). We observe that the Gabor representation explains brain activity in early visual areas for a large number of voxels such as V1, V2, and V3. The Gabor representation also explains brain activity consistently across subjects in the higher brain areas such as LO and precentral gyrus for 10 out of the 20 subjects. Similarly for the SIFT representation we observe that it explains brain activity in the lower visual areas such as V1, V2 and also higher areas of the brain such as LO across 9 out of 20 subjects. Overall Gabor and SIFT representations account for brain activity in similar areas of the brain. It is expected that Gabor and SIFT explain brain responses in early visual areas since both rely on edge filters. However, it is interesting to observe that they also explain brain activity in the higher areas of the brain.

We also observe areas where Gabor and SIFT together explain neural response consistently across subjects. The combination of Gabor and SIFT representations explain brain activity in 14 out of 20 subjects in the early visual area V1. The combination also explains brain activity in higher areas of the brain such as V4 and LO. This suggests that Gabor and SIFT representation have complementary low-level gradient information. Taken together, Gabor and SIFT provide a better computational basis for V1 representation.

### 3.4. Cross subject ROI analysis

**Figure 5:**
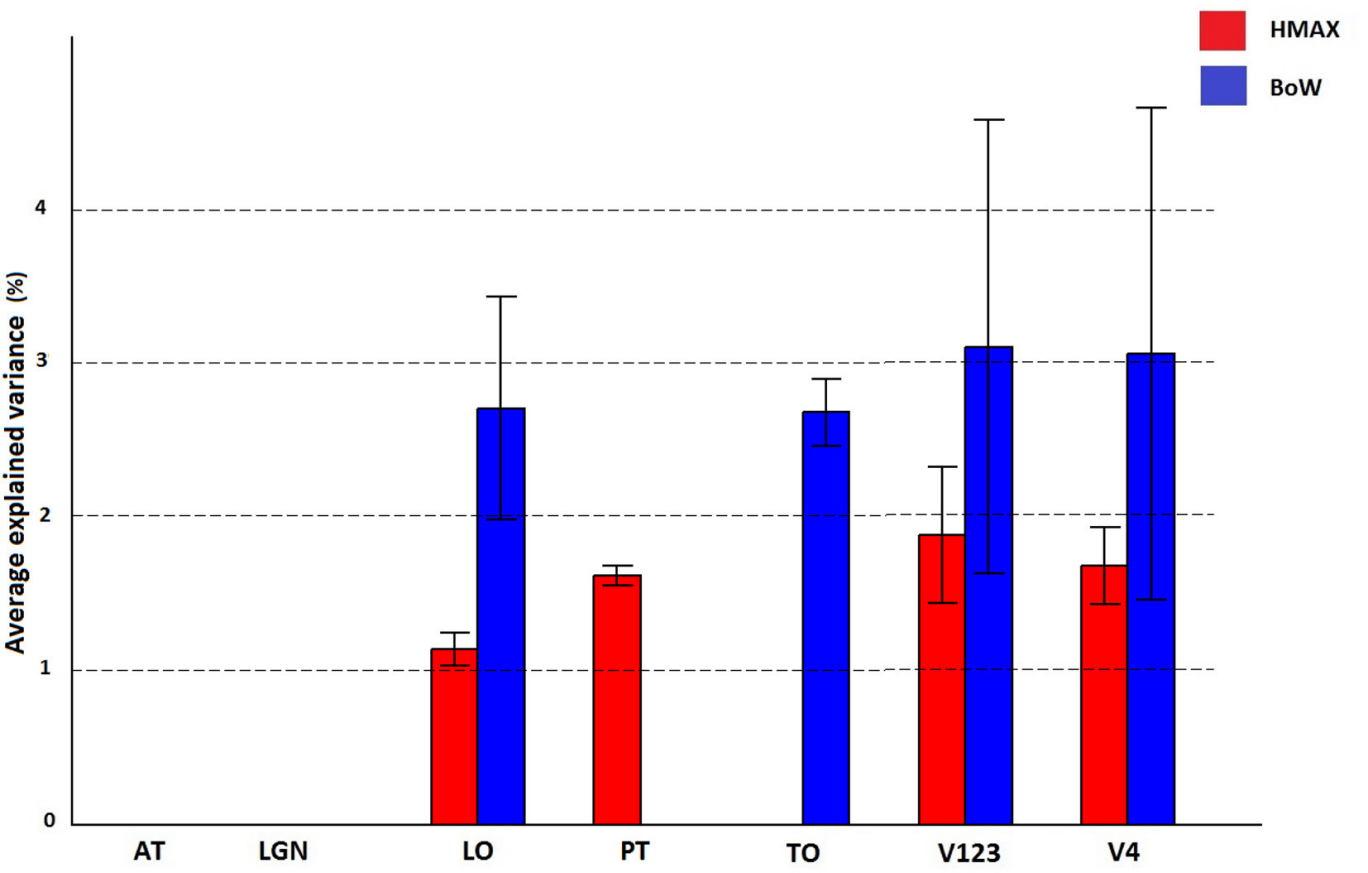
**Visualization of brain activity explained by HMAX and BoW model across all subjects in the selected ROIs**. The explained variation of the significant voxels (*p* < 0.05 and cluster size correction) are averaged across subjects over all voxels in a ROI.

A region of interest analysis was conducted to explicitly test the sensitivity of different brain regions to the models and their individual components. Figure 5 shows how HMAX and BoW explain brain activity in 6 brain regions (out of the 25 brain areas analyzed). These ROIs are obtained based on the Jülich MNI 2 mm atlas. We show the explained variation for each model averaged across subjects and the voxels within each ROI (Note that this doesn't show single subject variation across ROIs). We observe that there is significant explained variation in areas TO (temporal occipital), LO, explained variationV123 and V4. The representations do not account for brain activity in areas such as LGN (lateral geniculate nucleus) and AT (anterior temporal). In all the regions the BoW model has a higher average explained variation than the HMAX model The difference in explained variation is significant (*p* < 0.0001). Table 1 shows the number of voxels in each ROI obtained across subjects that exhibited significant brain activity and the maximum explained variation across subjects. We observe that the HMAX and BoW models explain more brain activity in early visual areas compared to the other areas.

**Table 1:** **Number of significant voxels and maximum explained variation for HMAX and Bow models in each ROI**.

	**No of significant voxels**		**Max explained variation**	
	**HMAX**	**BoW**	**HMAX**	**BoW**
AT	0	0	0	0
LGN	0	0	0	0
LO	43	387	2	4
TO	0	31	0	4
V123	701	3671	3	5
V4	53	1141	2	5

*The significant voxels (p < 0.05 and cluster size correction) are averaged across subjects over all voxels in a ROI*.

**Figure 6:**
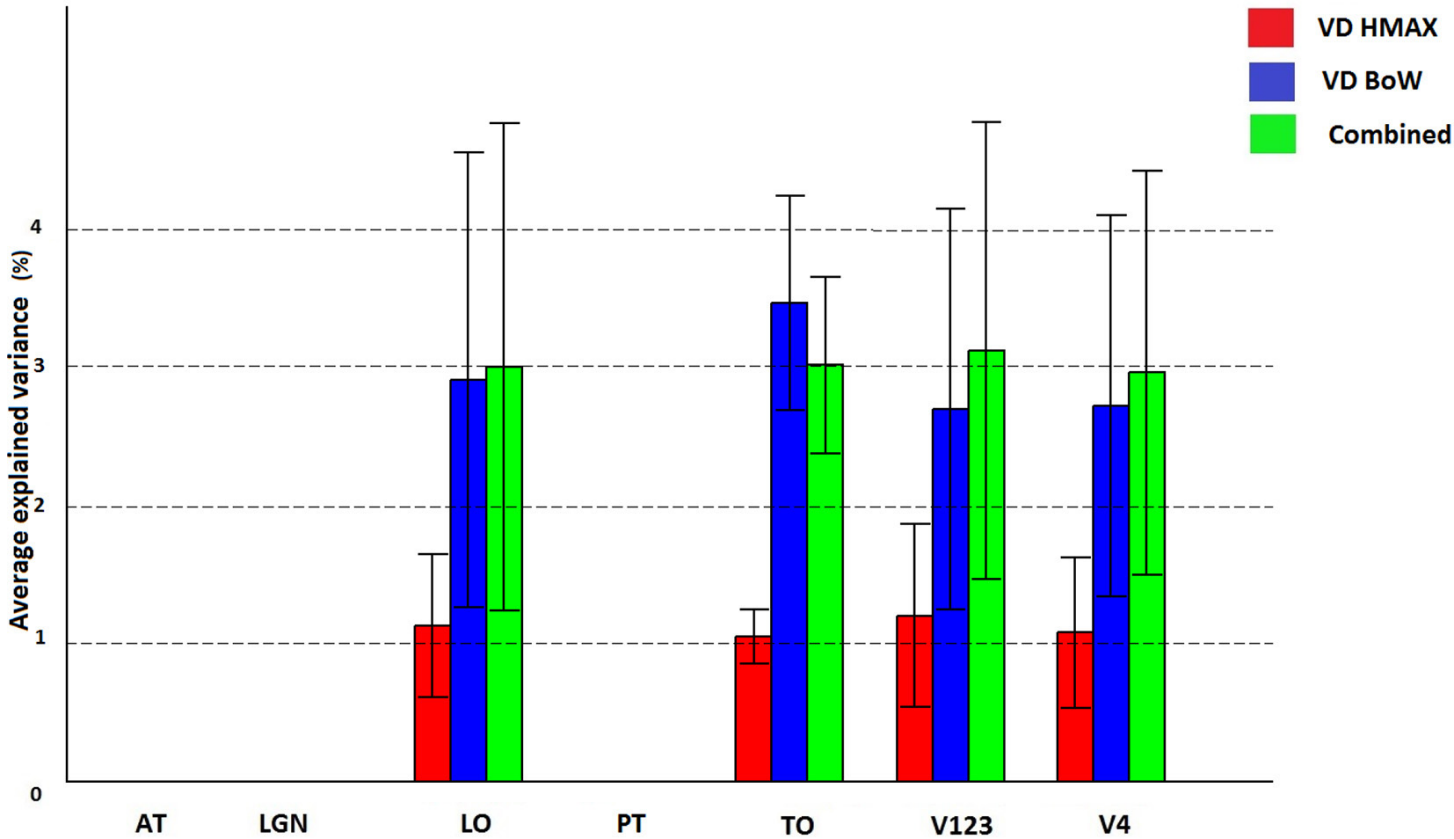
**Visualization of brain activity explained uniquely and the combination of visual dictionaries from HMAX, BoW across all subjects in the selected ROIs**. The explained variation of the significant voxels (*p* < 0.05 and cluster size correction) are averaged across subjects over all voxels in a ROI.

Figure 6 shows how visual dictionaries from HMAX and BoW explain brain activity in the 6 brain regions (out of the 25 brain areas analyzed). It can be seen that there is significant explained variation in areas TO (temporal occipital), LO, V123 and V4. The average explained variation is slightly higher in the TO regions compared to V123. In all the regions the visual dictionary from BoW model has a higher average explained variation compared to the visual dictionary from the HMAX model (*p* < 0.0001). Also the combination of visual dictionaries from HMAX and BoW do not significantly increase the explained variation and is similar to the explained variation from BoW. Table 2 shows that the visual dictionary from both the models explains a large number of voxels in LO and V4, however the visual dictionary from BoW has highest explained variations in LO and TO compared to HMAX. Also, we do not notice any brain activity in brain regions such as parahippocampal gyrus, retrosplenial corted and medial temporal lobe for either HMAX and BoW models.

**Table 2:** **Number of significant voxels and maximum explained variation for visual dictionaries from each ROI**.

	**No of significant voxels**			**Max explained variation**		
	**VD HMAX**	**VD BoW**	**Combined**	**VD HMAX**	**VD BoW**	**Combined**
AT	0	0	0	0	0	0
LGN	0	0	0	0	0	0
LO	1427	1255	1402	3	9	10
TO	178	143	164	2	11	8
V123	4818	5705	5716	3	6	6
V4	1682	1878	1910	2	6	6

*The significant voxels (p < 0.05 and cluster size correction) are averaged across subjects over all voxels in a ROI*.

**Figure 7:**
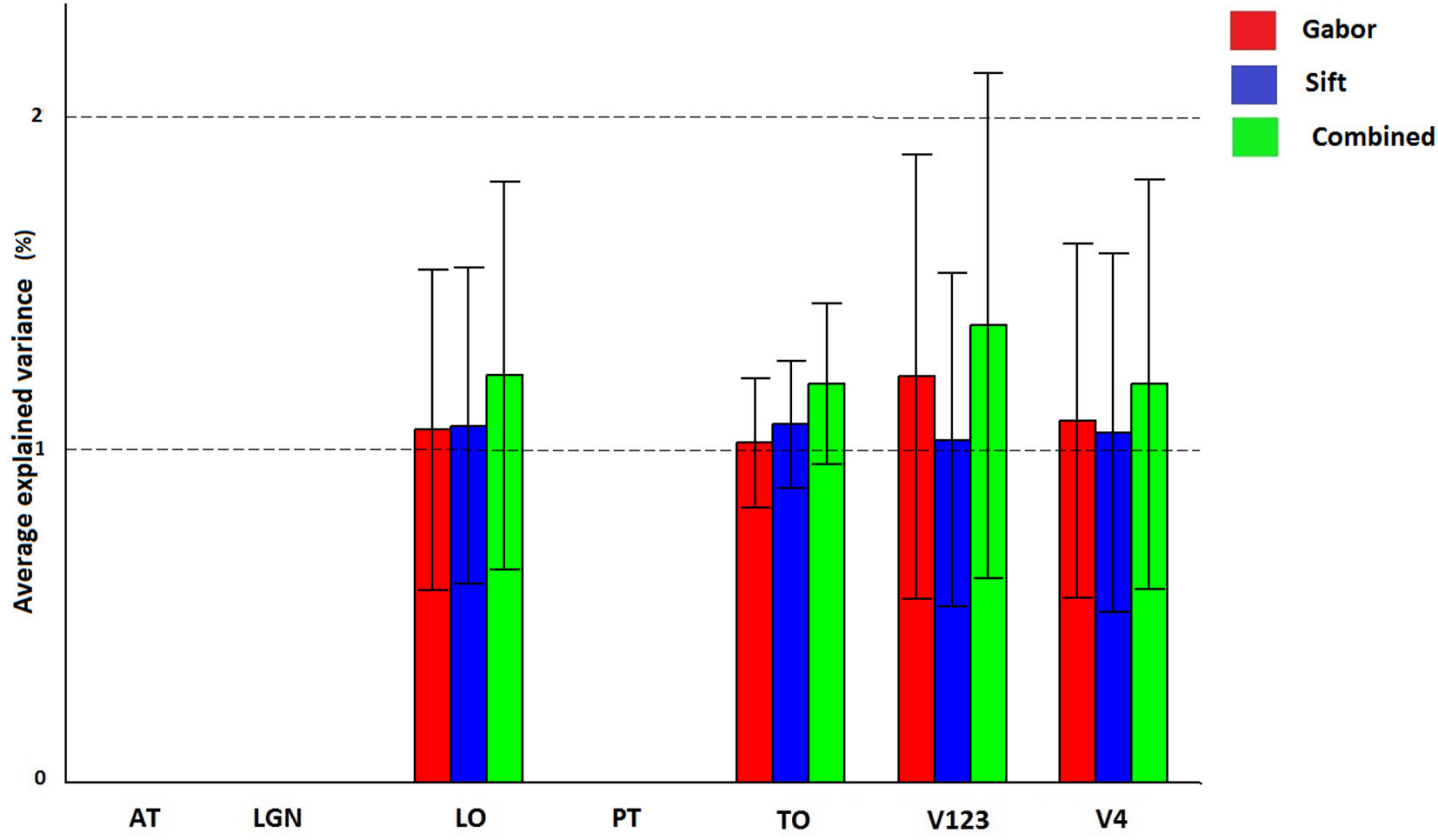
**Visualization of brain activity explained uniquely and the combination of Gabor and SIFT across all subjects in the selected ROIs**. The explained variation of the significant voxels (p < 0.05 and cluster size correction) are averaged across subjects over all voxels in a ROI.

Figure 7 and Table 3 show how Gabor and SIFT explain brain activity in the 6 brain regions (out of the 25 brain areas analyzed). We observe that there is significant explained variation in areas TO (temporal occipital), LO, V123 and V4. For Gabor, the average explained variation is slightly higher in the V123 region compared to the other areas. Here we also observe that the Gabor and SIFT representations are not significantly different from each other and also the combination explains brain acitivity to the same extent.

**Table 3:** **Number of significant voxels and maximum explained variation for each ROI**.

	**No of significant voxels**			**Max explained variation**		
	**Gabor**	**SIFT**	**Combined**	**Gabor**	**SIFT**	**Combined**
AT	0	0	0	0	0	0
LGN	0	0	0	0	0	0
LO	1394	1283	1427	2	2	2
TO	183	152	186	2	2	2
V123	5531	4418	5718	3	2	3
V4	1850	1535	1912	2	2	3

*The significant voxels (p < 0.05 and cluster size correction) are averaged across subjects over all voxels in a ROI*.

Overall these results suggest that individually, the BoW visual dictionary is a better computational representation of neural responses (as measured by percent explained variation and consistency across subjects) than the visual dictionary from HMAX, which provides little additional information over the BoW visual dictionary.

## 4. Discussion

The success of models such as HMAX and BoW can be attributed to their use of features of intermediate complexity. The BoW model in particular has proven capable of learning to distinguish visual objects from only five hundred labeled examples (for each category of twenty different categories) in a fully automatic fashion and with good recognition rates (Salimi-Khorshidi et al., 2014). Many variations of this model exists (Jégou et al., 2012), and the recognition performance on a wide range of visual scenes and objects, improves steadily year by year (Salimi-Khorshidi et al., 2014). The HMAX model is a biologically plausible model for object recognition in the visual cortex which follows the hierarchical feedforward nature of the human brain. Both the models are candidate computational models of intermediate visual processing in the brain.

Our results show that in early visual brain areas such as V1, V2, and V3 there are regions in which brain activity is explained consistently across subjects by both the HMAX and BoW models. These models rely on gradient information. In the HMAX model, Gabor filters similar to the receptive fields in the V1 region of the brain are at the basis of visual representation. Similarly in the BoW model, the Scale Invariant Feature Transform (SIFT) features are the low level representation based on multi-scale and multi-orientation gradient features (Lowe, 2004). Although SIFT features originate from computer vision, their inspiration goes back to Hubel and Wiesel's (1962) simple and complex receptive fields, and Fukushima's (1980) Neocognitron model. SIFT features thus have an embedding in the visual system, much like Gabor filters have. In light of this, the sensitivity in brain areas V1, V2, and V3 to representations of the HMAX and BoW models is natural and in part due to low-level features. Interestingly we also observe that SIFT and Gabor representations explain brain activity in higher regions of the brain. This indicates that neurons in higher level visual areas process low level features pooled over local patches of the image for feedforward or feedback processing within visual cortex.

Brain areas higher up in the processing hierarchy appear to be particularly sensitive to visual dictionaries. Visual dictionaries are medium size image patches that are informative and distinctive at the same time, allowing for sparse and intermediate representations of objects and scenes. In computer vision visual dictionaries have proven to be very effective for object and scene classification (Jégou et al., 2012). The brain may compute visual dictionaries as higher-level visual building blocks composed of slightly larger receptive fields, and use visual dictionaries as intermediate features to arrive at a higher-level representation of visual input. We observe that both HMAX and BoW visual dictionaries explain some brain activity in higher level visual regions, with the BoW visual dictionary representation outperforming the HMAX model both in terms of explained variance and consistency.

HMAX and BoW both use low level features that are pooled differently in the various stages of processing. First HMAX pools Gabor features by a local max operator whereas BoW creates a histogram of orientations (SIFT). Then, BoW uses a learning technique (k-means clustering) on all the SIFT features from the image to form the visual dictionary. On the other hand HMAX uses random samples of Gabor features pooled over patches as its visual dictionary. This difference in aggregating low level features might explain why BoW provides a better computational representation of images.

BoW visual dictionaries may facilitate scene gist perception, which occurs rapidly and early in visual processing. While there is evidence that simple low-level regularities such as spatial frequency (Schyns and Oliva, 1994), color (Oliva, 2000) and local edge aligment (Loschky and Larson, 2008) underly scene gist representation, it is hitherto unknown whether and how mid-level features facilitate scene gist perception. BoW summarizes SIFT features computed over the entire image. It has been observed that such patterns of orientations and scales are believed to be used by V4 and IT (Oliva and Torralba, 2006). This is in accordance with our observation that the localization of BoW visual dictionary representations occur in V4 and areas anterior to V4 in the brain.

Our findings are in line with a recent study by Leeds et al. (2013). They compared multiple vision models against MRI brain activity in response to image stimuli. Leeds et al. conclude that the BoW model explains most brain activity in intermediate areas of the brain. For this model, they report that the correlation of the BoW model varies from 0.1 to 0.15 across the 5 subjects. In our study, we obtain similar results for the BoW model, and with an average explained variation across subjects of around 5% (with explained variations varying across subjects). Similarities and consistencies between our results and results in Leeds et al. (2013) further suggest that BoW computation might provide a suitable basis for intermediate features in the brain. Yamins et al. (2014) observe explained variance of up 25% for both HMAX and BoW models, and up to 50% for their HMO model (4-layer Convolutional neural network model) in brain areas IT and V4. The discrepancy between these results and our findings in terms of the magnitude of explained brain activity can be in part attributed to the use of high signal-to-noise ratio measurements in Yamins et al. (2014), such as electrophysiological data from monkeys. The neural sensitivity to convolutional neural network model is nevertheless promising. We will include deep neural networks in future work to understand how it performs on video stimuli.

Our study aims to understand if intermediate features used in the brain are connected to how computational models of vision use such intermediate features. Our findings suggest that visual dictionaries used in HMAX and BoW account for brain activity consistently across subjects. The result does not imply that visual dictionaries as computed by HMAX or BoW are actually used by the brain to represent scenes but it does suggest visual dictionaries might capture aspects of intermediate features. The results from this work are similar to previous work and provides new interesting insights into the nature of intermediate features in the brain. We have also provided a novel framework which allows us to dissociate the different levels of a hierarchical model, and individually understand their contribution to explain brain activity.

### Conflict of interest statement

The authors declare that the research was conducted in the absence of any commercial or financial relationships that could be construed as a potential conflict of interest.
