# Supplementary material for: Visual dictionaries as intermediate features in the human brain
Source: Front Comput Neurosci. 2015 Jan 15;8:168. doi: 10.3389/fncom.2014.00168 (PMC4295527; doi:10.3389/fncom.2014.00168)
Supplement: Supplementary file 1 [file Image1.PDF]

1 SUPPLEMENTARY INFORMATION

1.1 INDIVIDUAL SUBJECTS

Using distance-based variation partitioning for each subject we dissociate the explained variation of the HMAX model into unique contributions of Gabor R^2_{gabor} and visual dictionary representation R^2_{vdhmax}. We do the same for the BoW model based on SIFT R^2_{sift} and visual dictionary representation R^2_{vdbow}. This resulted for each subject and for each model in two volumes indicating the strength and the significance of the explained variation. Cluster size correction ($p<0.05$ and minimal cluster size of 25 voxels) was performed to solve for the multiple comparison problem.

Figure 1 shows in the color red, areas and strengths of the unique contribution of the first hierarchical level of the models, Gabor and SIFT, in accounting for brain activity. It can be observed that for many subjects Gabor mainly explains brain activity in the higher visual areas such as Lateral occipital cortex, precuneous cortex, precentral gyrus. On average Gabor explains 4% of the variation in brain activity. For individual subjects the explained variation peaks at 8%. A similar pattern can be observed for SIFT: it mainly explains brain activity in higher areas involved in visual processing such as Lateral occipital cortex and middle temporal gyrus. SIFT has lesser average explained variation of 3%, and the explained variation peaks to 6% at the subject level.

The areas depicted in figure 1 in blue are associated with visual word representations. As can be observed the visual dictionaries from HMAX and BoW mainly account for activity in brain areas involved in early visual processing such as V1, V2, V3 and V4. Other brain areas such as LO and TO however also show activity that is explained by visual word dictionaries. While spatially the visual dictionary representations of HMAX and BoW display correspondence, the visual dictionary from HMAX explains on average up to 5% in areas V2-V3 against 18% in areas V3-V4 for the BoW model.

That visual word representations account for brain activity early in visual processing, and SIFT and Gabor representations at later stages is counterintuitive given that areas such as V1 are known to be sensitive to Gabor-like visual structures. It has to be noted, however, that we show only the unique contributions by Gabor, SIFT and they also share common explained variation with the visual dictionaries in the models in the early visual cortex.

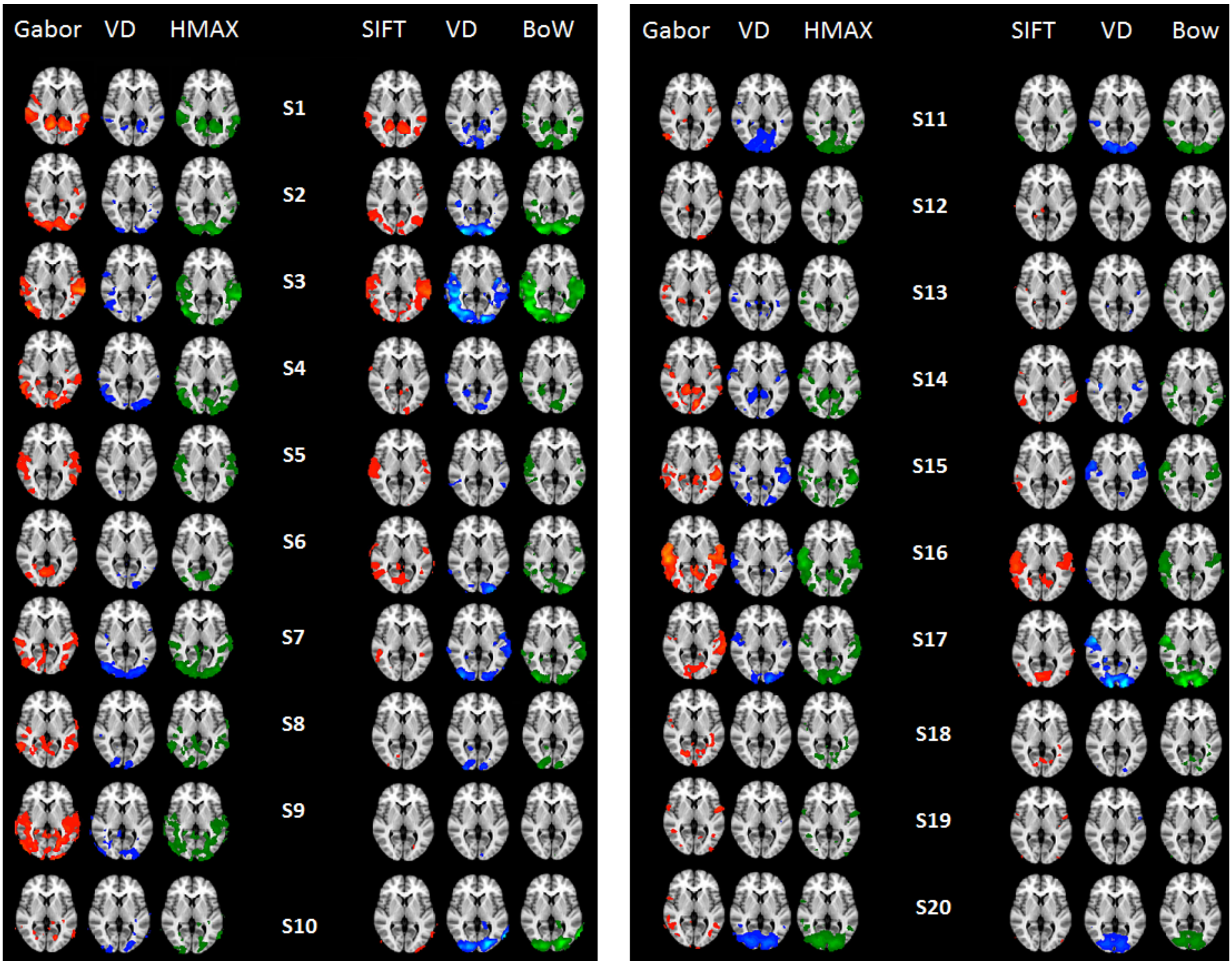

Figure 1: Variation partitioning on Y per individual subject to determine the fraction of explained variation Gabor(red) and visual dictionary (blue) from HMAX (green) model; SIFT (red) and visual dictionary (blue) from BoW (green) model. Visualization of the explained variations of only the significant voxels ($p < 0.05$ within a cluster size of 25) for the 20 subjects (S1 - S20).
